# Patients’ Experiences Following Osteoarticular Foot Surgery for Rheumatoid Arthritis-Related Deformities: A Qualitative Study

**DOI:** 10.3390/medicina61040677

**Published:** 2025-04-07

**Authors:** Amparo Campos-Cano, Ana Belen Ortega-Avila, Salvador Diaz-Miguel, Alejandro Castillo-Domínguez, Eva Lopezosa-Reca, Gabriel Gijon-Nogueron, Laura Ramos-Petersen, Andrés Reinoso-Cobo

**Affiliations:** 1 Department of Nursing and Podiatry, Faculty of Health Sciences, University of Malaga, 29016 Málaga, Spain; amparocampos@uma.es (A.C.-C.); anaortavi@uma.es (A.B.O.-A.); salvadordiazm@uma.es (S.D.-M.); alejandrocastillo@uma.es (A.C.-D.); evalopezosa@uma.es (E.L.-R.); gagijon@uma.es (G.G.-N.); andreicob@uma.es (A.R.-C.); 2 Biomedical Research Institute (IBIMA), 29010 Malaga, Spain

**Keywords:** rheumatoid arthritis, foot, patient experience, qualitative study, post- surgical outcomes

## Abstract

*Background and Objectives*: Rheumatoid arthritis (RA) is a chronic autoimmune disease that frequently causes foot deformities, decreasing mobility and quality of life. Although surgical interventions seek to alleviate these alterations, the long-term experiences of patients have not been deeply explored. The aim of this study was to describe the experiences of patients with RA undergoing osteoarticular surgery to correct acquired foot deformities. *Materials and Method*: A qualitative study design was used with structured interviews including 19 patients with RA treated in a specialised rheumatology service. The thematic analysis was carried out using the Braun and Clarke thematic analysis, ensuring compliance with ethical standards and the anonymity of the participants. *Results*: Five main themes were identified: experience with pain before and after surgery; impact on functional capacity; complications and need for additional surgeries; emotional impact and quality of life; overall satisfaction with the surgery. While many patients reported significant pain reduction and functional improvements, others faced recurrences of the deformities, persistent pain, and post-surgical complications. Emotional responses ranged from well-being to frustration, depending on surgical outcomes. The five-year follow-up period allowed for a comprehensive assessment of the long-term impact of surgery. The recurrence rate of deformities was notable, and the emotional impact of these recurrences was significant, with patients expressing frustration and distress in some cases. *Conclusions*: The patients’ experiences were heterogeneous, with both positive and negative outcomes. These findings underscore the importance of individualized management and comprehensive follow-up that consider the clinical outcomes, expectations, and emotional well-being of RA patients undergoing foot surgery.

## 1. Introduction

Rheumatoid arthritis (RA) is a chronic autoimmune disease [1]. Its usual presentation is characterised by joint inflammation, pain in the short term, destruction, and progressive osteoarticular deformation [2]. Foot deformation causes gait alteration [3], leading to functional deterioration [4] and a reduction in the quality of life [5]. Osteoarticular surgery should be considered as a treatment of choice for patients with RA, as it is a rheumatic disease with a prevalence of up to 90% of symptoms and deformity in the foot [6,7].

The management of RA includes both pharmacological treatments [8,9] and non-pharmacological interventions, such as health education [10], promotion of physical exercise [11], and orthopodiatric treatment [12]. When foot deformation limits the patient, osteoarticular surgical treatment is considered, aiming to improve foot functionality and reduce pain [13]. An individualised evaluation is essential, considering the structural deterioration of the foot and the adaptation of the pre- and post-surgical treatment, together with the level of activity of the disease [14].

In the current literature, different surgical methods with similar results are described, including arthrodesis of the hallux, resection of metatarsal heads (2nd to 5th), resection of all metatarsal heads (1st to 5th) [15], and conservation of metatarsal heads. The conservation of metatarsal heads is the most recommended [16,17], associated with new pharmacological treatments. Regardless of the technique, it is very important to carry out exhaustive control of the pre- and post-surgical antirheumatic treatment [18].

Although existing studies compare surgical techniques by assessing quality of life, functionality, and pain, they do not address long-term patient perceptions. This highlights the need for qualitative research, which captures emotional and personal aspects that quantitative methods cannot.

The role of nonsurgical interventions in this context has been insufficiently explored. However, recent reviews, such as the 2022 Cochrane Rehabilitation overview on the management of arthralgia in rheumatoid arthritis [19], highlight the importance of including nonsurgical treatments in the overall therapeutic strategy.

The current literature focuses mainly on comparing surgical techniques assessing quality of life, functionality, and pain, but lacks comprehensive analyses of long-term perceptions of patients. Specifically, there is limited research on the persistence of post-surgical symptoms, recurrence rates of deformities, and the emotional and psychological impact of these procedures over time. This study describes the experiences of patients in a five-year period after foot osteoarticular surgery, providing a perspective that complements the quantitative clinical data. The results will offer information of interest to personalise pre- and post-surgical management, thus improving comprehensive care focused on the specific needs of each patient.

The aim of the study was to describe the experiences of patients with RA undergoing osteoarticular surgery to correct acquired foot deformities.

## 2. Materials and Methods

### 2.1. Design

The present study used a qualitative approach to determine the long-term experiences of RA patients who underwent foot orthopaedic surgery. This qualitative design allowed us to understand the perspective of patients, analysing their experiences in detail. The present study followed an approach grounded in theory.

Although quantitative and mixed methods have their value, the qualitative approach was chosen for this study due to its ability to provide a deeper and more detailed understanding of the personal experiences of the patients, which is essential for assessing the long-term impact of surgical interventions in patients with chronic diseases such as RA.

### 2.2. Ethical Considerations

This study was carried out in full compliance with the provisions of the Declaration of Helsinki regarding ethical principles for medical research in human beings, with the authorization of the Ethics Committee of the University of Malaga (CEUMA-91-2015-H) and the Andalusian Health Service (PEIBA ARC0001).

### 2.3. Participants

The study sample was selected for convenience; all were patients diagnosed with RA from the Rheumatology Service of the Virgen de las Nieves University Hospital in Granada (Spain). Their referral rheumatologists invited participants to participate in the study. After accepting their participation, they were given the documentation explaining the methodology and objectives of the study and the written informed consent, which they signed.

Inclusion criteria: patients over 18 years of age and diagnosed with RA according to the 2010 Rheumatoid Arthritis Classification Criteria (endorsed by the American College of Rheumatology and the European League Against Rheumatism) [20]. Another inclusion criterion was that they had undergone their first foot osteoarticular surgery between 2017 and 2019, considering that the objective was to analyse their clinical evolution after 5 years.

Participants with difficulty communicating, expressing themselves, and answering questions asked in Spanish; patients with psychiatric comorbidities; and patients with surgery on their knee or hip were excluded.

The interviews took place between May and June 2024.

### 2.4. Data Collection

A structured interview was conducted with each patient individually, which had an average duration of 30 min. It was considered that the level of saturation of the questions was reached when the answers did not offer emerging themes in the new interviews, meaning that the themes were repeated in some interviews. A researcher (ARC) also reviewed the medical records of the patients to check the current clinical situation and the surgical processes that had been performed on each of the participants.

The interviews were conducted in the Rheumatology Service of the Virgen de las Nieves University Hospital (Spain), and a digital voice recorder was used to record them. At the same time, a researcher (ACC), with extensive podiatry experience, both in clinical and research areas, with patients diagnosed with RA, made notes that could enrich the message of the participant. To preserve anonymity, each patient was assigned a code. Only one member of the research team (data manager, GGN) had access to the correlation between the codes and the personal data of the participants. This encryption system ensured confidentiality, as that member managed the files using a password-protected system and was not involved in the data collection, thus avoiding any influence on the process.

The participants had to complete and sign the patient information sheet and informed consent. In addition, the researcher clarified any doubts that the participants had. Once the documentation was signed, the interview was carried out in a separate room to preserve privacy. All the information and documents obtained were kept in an office (to be stored for 5 years), to which only authorised members have access. Subsequently, all the information will be stored in the Department of Nursing and Podiatry of the University of Malaga in a space for documentation and exclusive information of studies carried out. These documents will be kept under lock and key for the next 15 years. At the end of the period, they will be destroyed.

The interview questions were written based on the West Haven Yale Multidimensional Pain Inventory (WHYMPI) [21], taking as a reference a previous review of the current literature on patients with RA and foot osteoarticular surgery. The research question of the study was as follows: what are the experiences of patients with RA who, due to their clinical situation, undergo foot osteoarticular surgery? And the following questions were generated:

1. Remembering what your symptoms were like prior to foot surgery, could you explain what the foot pain was like and what limitations it generated?
2. After foot surgery, how has your experience with pain? Do you feel less pain compared to before the operation?
3. After surgery, do you experience pain at rest, when walking, or both?
4. Have you noticed any differences in your ability to work, perform household chores, or other activities that require constant standing or moving after foot surgery?
5. Have you experienced any type of limitation or restriction in your daily activity since surgery?
6. What impact has foot surgery had on your mood, emotions, and overall well-being?
7. Has there been a particular activity that you couldn’t do before because of your feet that you can now do after surgery?
8. Have you experienced any complications since surgery (infection, healing issues, etc.)?
9. Have you had to undergo additional surgery after the first surgery due to any complications?
10. Overall, how would you describe your satisfaction with foot surgery as part of your treatment for rheumatoid arthritis? Has it been a positive experience for you?

It should be added that the participants did not have access to the conversations in digital format or their transcription, nor were the interviews repeated with the aim of completing or replacing the answers.

Forefoot deformation was assessed by two expert foot and ankle researchers (ACC and ARC) using the Manchester Hallux Valgus scales to assess the degree of deformation of the first ray [22] and the Nijmegen scale to estimate the anatomical changes that occur in the lesser toes [23].

Also, some characteristics of the participants were collected: sex, height, weight, age, years of evolution in RA, and specific treatment of RA.

Techniques to enhance trustworthiness and credibility of data analysis were carried out, such as recording the interviews, collecting notes, and interviewing participants with different points of view.

### 2.5. Data Analysis

The qualitative analysis of the interviews was conducted by two researchers (LRP and ARC) using the Braun and Clarke framework, which was chosen for its flexibility in identifying patterns in the data and for its ability to provide a detailed interpretation of the experiences of the participants compared to other methods [24]. The transcribed interviews of each participant were analysed line by line to generate initial codes. These codes were organized and grouped into the main themes. Both the frequency of the emerging codes and their relevance of the information to the participants were considered for their classification. To ensure the validity and reliability of the results, the data were independently coded by two researchers. After the initial coding, consensus meetings were held to discuss discrepancies and reach an agreement on categorisation and final themes. The coded fragments were evaluated, defined, and classified within their respective themes. To facilitate the coding and analysis process, MAXQDA software was used. Finally, the results were reviewed and validated by the co-authors.

## 3. Results

A total of 19 interviews were analysed thematically. One of the participants was male and eighteen were female, with a mean age of 64.9 ± 11.8 years and with 21.7 ± 11.6 years of evolution of the disease. Their average height was 159.3 ± 6 cm and weight 68.6 ± 15.9 kg.

Regarding pharmacological treatment, 7 out of 19 of the participants (36.8%) received methotrexate, 11 out of 19 participants (57.89%) were on biological therapy, and 1 out of 19 participants (5.2%) followed a combined treatment of methotrexate and biological therapy. Furthermore, 8 out of 19 participants (42.1%), regardless of their treatment, were also taking corticosteroids.

In terms of the foot assessment, especially their first ray assessment according to the Manchester Hallux Valgus scale [23], 6 out of 19 participants (31.57%) were classified with grade A, 7 out of 19 participants (36.8%) with grade B, 5 out of 19 participants (26.43%) with grade C, and 1 out of 19 participants (5.2%) with grade D. Assessing their lesser toes according to the Nijmegen scale [22], 2 out of 19 participants (10.52%) presented a classification of 0, 7 out of 19 participants (36.8%) of 1, 6 out of 19 participants (31.57%) of 2A and 2B, and 4 out of 19 participants (21.11%) of 3A and 3B.

In the forefoot, 12 out of 19 patients (63.15%) underwent surgery for HAV and 10 out of 19 patients (52.63%) for lesser toes, 2 out of 19 patients (10.52%) underwent surgery on the hindfoot, and no patients underwent midfoot surgery. It is important to emphasize that 8 out of 19 patients (42%) experienced recurrence of the operated deformity, a finding of great clinical relevance that underscores the importance of addressing potential post-surgical complications, being 1 out of 2 patients (50%) of those operated on the hindfoot and 9 out of 12 patients (75%) on the forefoot. Also, one participant had an extra operation to resolve Hallux Varus.

### 3.1. Qualitative Analysis

A total of 67 codes were identified, which were then organized into five themes. The resulting themes were agreed upon by the authors to improve the validity of the data (Table 1):

- Experience with pain before and after surgery.
- Impact on functional capacity.
- Complications and need for additional surgeries.
- Emotional impact and quality of life.
- Overall satisfaction with the surgery.

#### 3.1.1. Experience with Pain Before and After Surgery

Prior to surgery, some patients perceived severe, persistent, and limiting pain that severely affected their quality of life and mobility.

“It hurt a lot, I had claw toes and I couldn’t walk with shoes on.” (PAC001)

“The pain was unbearable, walking like a robot.” (PAC010)

Regarding post-surgery pain, different experiences were described, which varied from complete elimination (especially those with previous severe deformities) to the persistence of severe symptoms in one or both limbs. In addition, some participants indicated that when the pain returned over time, it was often associated with new deformities, such as hammertoes.

“I don’t have any pain, I’m great.” (PAC005)

“I feel less pain, but the toes have been deformed.” (PAC019)

“The right foot was perfect, but the left still hurts.” (PAC008)

#### 3.1.2. Impact on Functional Capacity

The surgery significantly affected the functional capacity of the participants, with mixed results obtained. In some cases, patients regained partial or full mobility, allowing them to resume activities such as walking long distances, performing household chores, and participating in recreational activities. However, others noted that they continued to experience physical limitations, either from residual pain, persistent deformities, or a lack of stability in their feet. These limitations led to a reliance on external supports, such as crutches or specialized footwear, and in some cases, the need for third-party support to perform everyday tasks.

“Now I live a normal life, I walk 6 or 7 kilometres a day, I swim.” (PAC005)

“I can’t wear any kind of shoe, I have to be barefoot.” (PAC007)

“Climbing stairs is hard because I don’t have mobility in my ankle.” (PAC002)

“I need help for the house, I have a hard time climbing stairs.” (PAC009)

#### 3.1.3. Complications and Need for Additional Surgeries

A considerable number of participants reported post-surgical complications, such as infections, stiff toes, and recurrence of deformities. In some cases, these complications require additional interventions, resulting in a significant emotional and physical impact. For others, fear or negative experiences associated with surgery led them to avoid further operations, even when recommended by doctors. Although several participants expressed that they did not experience major complications, unmet expectations regarding the results of the surgery also generated dissatisfaction.

“I had to have a second surgery because the bunion.” (PAC003)

“I was hospitalised for 21 days for an infection after the operation.” (PAC018)

“After the surgery I had a stroke and I was afraid to have another operation.” (PAC008)

“I don’t want to have another operation because my foot could be worse.” (PAC004)

#### 3.1.4. Emotional Impact and Quality of Life

Post-surgery moods and well-being ranged from optimism to frustration. In cases where they reported improved mood, it was due to a reduction in pain owing to the satisfaction of the surgery. These noted an increase in their confidence and overall well-being. Some even highlighted the ability to return to activities that were previously impossible for them.

“After surgery I feel much better because I can walk more.” (PAC003)

“Now I can wear sandals and show my feet without hesitation.” (PAC017)

On the other hand, participants who experienced discouragement indicated that it was due to frustration with complications from surgery or recurrent deformities. Also, in some cases, it was due to complexes associated with the appearance of their feet. This contrast shows how the success of surgery directly affects the psychological well-being of patients.

“It creates a lot of problems for me; the feet are very deformed.” (PAC014)

“At first I was happier, but I’m still the same because the toes were deformed again.” (PAC019)

#### 3.1.5. Overall Satisfaction with Surgery

Satisfaction is totally linked to the result after the surgery. Some patients described it as a very positive experience, highlighting functional and aesthetic improvements that restored quality of life. In these cases, the improvements were tangible and sustained.

“For me it has been very positive, I give it a 10.” (PAC005)

“Each case is different, but I have noticed improvement.” (PAC004)

On the other hand, some participants expressed dissatisfaction, claiming a lack of improvements, recurrence of deformities, or adverse side effects. In general, the perception of success was mediated by the relationship between expectations and obtained results, as well as by the evolution of the underlying disease.

“If I could go back, I wouldn’t have surgery; the pain continues.” (PAC018)

“In general, I am not satisfied; I’m pretty much the same as I was before I had surgery.” (PAC019)

## 4. Discussion

The aim of the study was to describe the experiences of patients with RA undergoing osteoarticular surgery to correct acquired foot deformities. The results offer a vision from the point of view of the patient with RA in relation to foot surgical treatment, which aims to resolve the deformity acquired with the evolution of the disease. The patients were able to openly describe their experiences, the benefits, and the difficulties they had in their daily lives in the five years following their foot surgery.

Recent evidence supports the use of psychological interventions such as cognitive-behavioural therapy (CBT) in managing chronic pain and post-surgical emotional distress. CBT has shown benefits in improving coping strategies, reducing anxiety, and enhancing recovery in patients undergoing surgery for chronic inflammatory conditions [25].

In general, with the analysis of the interviews carried out, patients expressed improvements in pain and functionality, but it should be noted that some patients, in their experiences, emphasised that they had not met their expectations, even showing a rejection of this therapeutic option if they could go back. These findings may be related not only to structural complications, but also to central pain mechanisms and sensitization processes, as highlighted in the recent literature on osteoarthritis [26]. Moreover, assessments of functionality such as pinch strength have demonstrated the value of reliable physical evaluation tools in chronic joint conditions [27].

The patients described a significant decrease in their foot pain, particularly patients with severe HAV deformities and claw toes, which is consistent with previous studies. Fukushi et al. reported excellent results with significant pain reduction with 28-month follow-up [28]. We must also highlight a significant proportion of patients, with persistence and even increased pain in the results obtained, agreeing with the study by Yuan He et al. who referred to post-surgical pain and associated it with plantar keratosis in pressure regions and increased deformation of both first ray and lesser toes [15]. Despite advances in surgical procedures and pharmacological management, the persistence of complications and recurrence rates remain high. This may be due to the chronic and progressive nature of RA, advanced anatomical deterioration at the time of surgery, limitations in post-surgical rehabilitation adherence, or insufficient integration of multidisciplinary approaches.

Patients emphasised foot functionality, showing improvements and limitations, similar to the results of Sawachica et al. [13]. Based on the results of the present study, these can be categorised into two subgroups: in the first subgroup, the participants highlighted significant improvements, and in the other, they reported that the surgery was not resolutive, maintaining or increasing its limitations. Aletaha et al. associated the loss of functionality with aging and the years of evolution of the disease, considering it as an irreversible factor [29]. These two factors may have probably influenced the results of the present study, since foot deformation in RA is directly associated with years of evolution.

A relevant aspect is the high incidence of recurrence, which is similar to that described in the reviewed literature [28,30]. These complications led patients to undergo further surgery or accept non-resolutive surgery. In some cases, even more severe iatrogenic deformities were described. This finding highlights the importance of implementing personalized postoperative follow-up in patients with RA.

Another finding to highlight is the high incidence of post-surgical complications such as infections and joint stiffness, among others, with prevalences similar to those in other studies [31]. Reeves et al. associated it with vascular complications, healing difficulties, degree of foot deformation, and years of evolution, recommending optimal perioperative therapeutic management to minimise it [32].

The experiences of the participants in this study show radically opposite situations, identifying patients who reported having recovered activities that they could not perform before the intervention and another group of patients who considered that the surgery caused frustration and discouragement, associating it with recurrent deformities and increased pain. A progressive loss of quality of life related to loss of function and pain is described in a different study [5], also highlighting social isolation, as the patients are unable to carry out their usual activities with their family and friends [33].

The strengths of this study should be highlighted: firstly, it provides valuable insights through the experiences of patients with long-term RA and the impact it has on their lives. The experience of the research team made up of expert podiatrists in rheumatology facilitated the interpretation of the data obtained, and it should also be noted that the selection of participants was carried out by their reference rheumatologists.

Although the study provides valuable insights, it is necessary to identify the inherent limitations of its qualitative design. Focusing our results on the experiences of patients and not contemplating that of professionals, and the lack of a longitudinal follow-up, limit us in generalising the findings. Moreover, the study’s single-centre design must be recognized, as it limits the ability to generalise the findings to a broader population. It is important to note that convenience sampling may have introduced selection bias, as the sample may not have been representative of the general RA patient population, potentially lacking diversity in terms of socioeconomic, cultural, and clinical characteristics. This limits the ability to generalise the results to a broader population. Furthermore, the lack of participant validation of the transcripts represents a limitation, as it could have offered an opportunity to confirm the accuracy and reliability of the data. Additionally, the influence of interviewer bias must be considered when interpreting the data.

## 5. Conclusions

The patients’ experiences were heterogeneous, showing both positive and negative outcomes. The study highlights the diversity of the experiences of RA patients undergoing surgery to correct foot deformities. Not all patients reported positive results; approximately half expressed postoperative complications, persistence or increased pain, and functional limitations. These findings emphasise the variability in the experiences of the patients, showcasing how individual differences in pain, recovery, and satisfaction can significantly impact overall outcomes. The present results underscore the importance of careful assessment, personalized follow-up, and comprehensive strategies, underscoring the need for an integrated approach involving specialists such as podiatrists, rheumatologists, physiotherapists, and psychologists. To improve clinical practice, it is essential to implement individualized care plans that address each patient’s specific needs and expectations. This may include the regular monitoring of pain levels, functional progress, and emotional well-being, along with targeted interventions to address postoperative complications and optimize recovery. To optimize post-surgical outcomes and quality of life, it is crucial to incorporate customized orthoses, structured rehabilitation, and psychological support. Additionally, prioritizing shared decision-making and individualized follow-up plans is essential to aligning interventions with patients’ expectations, promoting optimal recovery and functional outcomes.

## Figures and Tables

**Table 1:** Key quotes for each theme.

Theme	Key Quotes
Experience with pain before and after surgery	“It hurt a lot, I had claw toes and I couldn’t walk with shoes on.” (PAC001)“The pain was unbearable, walking like a robot.” (PAC010)“I don’t have any pain, I’m great.” (PAC005) “I feel less pain, but the toes have been deformed.” (PAC019)
Impact on functional capacity	“Now I live a normal life, I walk 6 or 7 km a day, I swim.” (PAC005) “I can’t wear any kind of shoe, I have to be barefoot.” (PAC007) “Climbing stairs is hard because I don’t have mobility in my ankle.” (PAC002) “I need help for the house, I have a hard time climbing stairs.” (PAC009)
Complications and need for additional surgeries	“I had to have a second surgery because of the bunion.” (PAC003)“I was hospitalized for 21 days for an infection after the operation.” (PAC018)“After the surgery I had a stroke and I was afraid to have another operation.” (PAC008)“I don’t want to have another operation because my foot could be worse.” (PAC004)
Emotional impact and quality of life	“After surgery I feel much better because I can walk more.” (PAC003) “Now I can wear sandals and show my feet without hesitation.” (PAC017) “It creates a lot of problems for me; the feet are very deformed.” (PAC014) “At first I was happier, but I’m still the same because the toes were deformed again.” (PAC019)
Overall satisfaction with surgery	“For me it has been very positive, I give it a 10.” (PAC005) “Each case is different, but I have noticed improvement.” (PAC004) “If I could go back, I wouldn’t have surgery; the pain continues.” (PAC018) “In general, I am not satisfied; I’m pretty much the same as I was before I had surgery.” (PAC019)

## Data Availability

The original contributions presented in this study are included in the article. Further inquiries can be directed to the corresponding author.

## Abbreviations

The following abbreviations are used in this manuscript:

RA	Rheumatoid arthritis
CEUMA	Ethics Committee of the University of Malaga
WHYMPI	West Haven Yale Multidimensional Pain Inventory
